# Bone callus formation is highly disrupted by dietary restriction in growing rats sustaining a femoral fracture^1^

**DOI:** 10.1590/s0102-865020190010000002

**Published:** 2019-02-14

**Authors:** Iara Inácio Botega, Ariane Zamarioli, Patrícia Madalena San Gregório Guedes, Raquel Assed Bezerra da Silva, João Paulo Mardegan Issa, Mariana Maloste Butezloff, Yara Terezinha Corrêa Silva Sousa, João Paulo Bianchi Ximenez, José Batista Volpon

**Affiliations:** I Fellow Master degree, Postgraduate Program in Health Sciences Applied to the Locomotor System, School of Medicine, Universidade de São Paulo (USP), Ribeirao Preto-SP, Brazil. Design of the study, technical procedures, acquisition and interpretation of data, manuscript preparation.; II Researcher, Laboratory of Bioengineering, School of Medicine, USP, Ribeirao Preto-SP, Brazil. Design of the study, interpretation of data, manuscript preparation, critical revision.; III Fellow Master degree, Postgraduate Program in Health Sciences Applied to the Locomotor System, School of Medicine, USP, Ribeirao Preto-SP, Brazil. Technical procedures, acquisition of data.; IV PhD, Associate Professor, Department of Children’s Clinic, School of Dentistry, USP, Ribeirao Preto-SP, Brazil. Technical procedures, critical revision.; V PhD, Associate Professor, Department of Morphology, Physiology and Basic Pathology, School of Dentistry, USP, Ribeirao Preto-SP, Brazil. Technical procedures, critical revision.; VI Fellow PhD degree, Postgraduate Program in Health Sciences Applied to the Locomotor System, School of Medicine, USP, Ribeirao Preto-SP, Brazil. Technical procedures.; VII PhD, School of Dentistry, Universidade de Ribeirão Preto (UNAERP), Brazil. Technical procedures, critical revision.; VIII Fellow PhD degree, Postgraduate Program in Toxicology, School of Pharmaceutical Sciences, USP, Ribeirao Preto-SP, Brazil. Statistical analysis, technical procedures, critical revision.; IX Full Professor, Department of Biomechanics, Medicine and Rehabilitation of the Locomotor System, School of Medicine, USP, Ribeirao Preto-SP, Brazil. Design, intellectual and scientific content of the study; manuscript preparation, critical revision, final approval.

**Keywords:** Malnutrition, Osteoporosis, Fractures, Bone, Bony Callus, Rats

## Abstract

**Purpose:**

To evaluate the effects of food restriction on fracture healing in growing rats.

**Methods:**

Sixty-eight male Wistar rats were assigned to two groups: (1) Control and (2) Dietary restriction. After weaning the dietary restricted animals were fed ad libitum for 42 days with 50% of the standard chow ingested by the control group. Subsequently, the animals underwent bone fracture at the diaphysis of the right femur, followed by surgical stabilization of bone fragments. On days 14 and 28 post-fracture, the rats were euthanized, and the fractured femurs were dissected, the callus was analyzed by dual-energy X-ray absorptiometry, micro-computed tomography, histomorphometry, mechanical tests, and gene expression.

**Results:**

Dietary restriction decreased body mass gain and resulted in several phenotypic changes at the bone callus (a delay in cell proliferation and differentiation, lower rate of newly formed bone and collagen deposition, reductions in bone callus density and size, decrease in tridimensional callus volume, deterioration in microstructure, and reduction in bone callus strength), together with the downregulated expression of osteoblast-related genes.

**Conclusion:**

Dietary restriction had detrimental effects on osseous healing, with a healing delay and a lower quality of bone callus formation.

## Introduction

 Several factors are known to play a central role in maximizing skeletal acquisition, which is of great importance as this increases both bone mass and quality, and thus, reduces the risk of fractures and incidence of osteoporosis later in life^1^. Epidemiologic data indicate that a 10% increase in peak bone mass may decrease the risk of fracture^2^. Conversely, low bone mass in young adults represents a substantial risk factor for postmenopausal osteoporosis^3^. Thus, genetic inheritance, good health, and adequate nutritional intake are critical factors in optimizing bone mass accrual during skeletal maturation^1^ ^,^ ^4^. Conversely, it has been shown in rats that caloric restriction during rapid skeletal growth is detrimental to bone mass and architecture^1^.

 The term malnutrition refers to deficiencies, excess, or imbalances in an individual’s food intake. Specific micronutrient-related malnutrition refers to the lack of nutrients, while the term undernutrition means a general reduction in nutrient intake. Considering the relationship between dietary restriction and loss of bone quality^1^ ^,^ ^5^ ^-^ ^8^ associated with the fact that an adequate normal bone microenvironment is needed for fractures to heal properly, it is not surprising that undernutrition may result in impaired bone reparation. Previous authors have documented a disruption in fracture healing in rats submitted to dietary restriction of specific nutrients such as vitamin D or proteins^9^ ^-^ ^14^. However, clinical undernutrition exhibited by many patients may be caused by a reduction in total food intake instead of the depletion of specific nutrients^8^. To our knowledge, this study is the first to be conducted in growing rats fed with general diet restriction and sustaining a bone fracture. Undernutrition is widespread across different countries and mainly among poor people, which includes a significant proportion of children. The primary mechanisms underlying undernutrition and disruption of bone healing are yet to be determined. However, several pathways have been proposed to explain the delayed endochondral bone formation observed in animals restricted to specific nutrients, including poor cellular proliferation with the callus containing a preponderance of undifferentiated tissue and much less cartilage and bone, probably due to reduced levels of growth factors (i.e., insulin growth factor [IGF])^11^ ^,^ ^15^. 

 The aim of this study was to investigate the effects of ordinary dietary restriction on bone healing of growing rats. We hypothesized that undernutrition delays bone healing by decreasing bone cell differentiation and expression and by dissociating bone formation. Thus, we believe that food restriction may down-regulate the expression of genes related to osteoblastogenesis and reduce collagen deposition thus impairing the fracture callus.

## Methods

###  Animals and experimental groups 

 The Institutional Animal Care and Use Committee of our Institution approved all the animal procedures described in this study (Protocol 013/2016). 

 After weaning (21-day-old), male Wistar rats were housed in metabolic cages to monitor daily food consumption. A 3-day period was allowed for adaptation to the laboratory environment (controlled conditions of humidity, temperature (23±1°C), and an artificial light/dark cycle of 12 hours). 

 A total of 68 rats were divided into two groups (n=34 per group): (1) Con: weight-matched control rats with unlimited access to standard diet and (2) Res: rats fed a 50% of the *ad libitum* food intake of the control group. All animals had free access to water. The 50% dietary restriction protocol^16^ was calculated by measuring the daily food ingested by control rats. Forty-two days later animals of both groups underwent a closed femoral fracture and were separated into two subgroups according to the post-operative follow-up: 14 and 28 days (n=20 and 14, respectively). The different times for the end-point analysis represented two distinct stages in physiological fracture healing in rats: soft and hard bone callus formation, respectively^17^ ^,^ ^18^. 

###  Closed femoral fracture 

**Figure 1:**
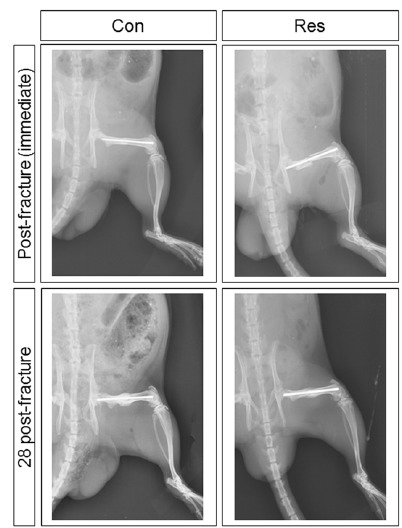
Immediate lateral X-rays of the pelvic limb of a rat following fracture fixation and 28 days post-fracture. The fragments are well aligned and the intramedullary rod is well placed. Con: control group; Res: dietary restriction group.

 Anesthesia was carried out with a solution (1:1) of xylazine and ketamine (0.1 mg/100 g) injected intramuscularly into the left gluteus maximus and a bone fracture was produced at the mid-diaphysis of the right femur^19^. To this aim, the right rat’s thigh was shaved and then placed on two metallic supports. A blunt blade controlled by a lever, aiming at the mid thigh was lowered and forced until an abrupt decrease in resistance occurred, thus indicating the bone failure^19^. Afterwards, the whole pelvic limb was cleansed with a 0.5% alcohol-based chlorhexidine solution, and under the common surgical environment, an approximately 1.0-cm long incision was made on the lateral surface of the mid-thigh over the fracture. The incision was extended along the intermuscular septum until the fractured bone was reached, whose extremities were minimally exposed and inspected. Animals with fractures not located at the shaft region or with more than three fragments (comminution) were discarded. Subsequently, a 1.0-mm thick orthopedic Kirschner wire (K-wire) was introduced into the medullary canal of the proximal fragment until it protruded through the skin at the trochanteric region. The fracture was reduced under direct vision and stabilized by advancing the K-wire into the medullary canal of the distal fragment until the femoral condyles were reached. The excess length of the K-wire that protruded from the trochanteric region was sectioned at the greater trochanter tip. The wound tissue was closed in layers with a 4-0 absorbable suture (Monocryl, Ethicon, USA), and the skin closure was sprayed with a solution (Adestro, Copelli, Brazil) to avoid self-mutilation. Immediately after surgery, the X-rays showed that the fragments were well aligned and fixed (Fig. 1). The X-rays taken immediately after the euthanasia (anteroposterior and profile views) showed the maintenance of the fixation and the callus development.

 Dipyrone solution (80 mL diluted 1:5 in saline) was used as an analgesic and injected subcutaneously, before the operation and then every 12 hours for five days. All animals were examined daily to verify the general health, cage activity, weight bearing, wound appearance, swelling, and range of motion of the knee and hip. The animals were weighed three times weekly.

###  Euthanasia 

 On days 14 or 28 post-fracture (11 weeks old and 13 weeks old, respectively) rats were euthanized with an overdose of sodium thiopental (Tiopental^®^, Cristália, Brazil) injected intraperitoneally. The fractured femurs were collected, the K-wires were removed, and the bone was cleaned of soft tissue with care not to disturb the callus envelope. The specimens reserved for mechanical analysis, densitometry, and micro-computed tomography (μCT) were kept in 70% ethanol solution and the specimens for histology studies were fixed in cold 4% paraformaldehyde.

###  Bone densitometry by Dual-energy X-ray Absorptiometry 

 Bone densitometry was assessed by Dual-energy X-ray Absorptiometry *(*DXA) with a Lunar DPX-IQ densitometer (*Lunar; software version* 4.7e, GE Healthcare, United Kingdom). After scanning the entire bone, the callus area was selected as the region of interest (ROI) to determine its bone mineral density (BMD) and bone mineral content (BMC). The scanning reproducibility (4.5%) was assessed by the root mean square coefficient of variation^20^.

###  Bone microstructure assessment by micro-computed tomography 

 After DXA assessment, femurs (n=7 per group) were scanned using a μCT device (SkyScan 1176; Bruker-microCT, Kontich, Belgium), at 65 kV, using a 1-mm-thick aluminum filter, a 360° rotation step of 0.4°, one-frame averaging, and an isotropic resolution of 8.5 μm. The reconstruction of images was performed using specific software (NRecon v.1.6.9). The whole callus was established as the region of interest (ROI). The fracture callus was analyzed using CT scan software (CTAn v.1.15.4) to determine the total callus volume (CV, in mm^3^) and the connectivity density among trabeculae forming the callus (ConnD, in 1/mm^3^)^20^.

###  Histological analysis and collagen deposition assessment 

 Histological analysis was carried out in seven femurs from each group. The bones were fixed in cold 4% paraformaldehyde, decalcified in cold 10% EDTA, embedded in paraffin, and 5 μm coronal semi-serial sections were obtained. From each specimen, after collecting twelve sections, the subsequent ten sections were discarded; this procedure was repeated for the whole callus. The sections were stained with hematoxylin and eosin (HE), Masson’s trichrome, or picrosirius red, and analyzed under bright field microscopy (Axiovert; Carl Zeiss, Germany). Images were captured with a CCD camera (AxioCam MRc; Carl Zeiss, Germany) with magnifications of 12.5×, 50×, and 100×.

###  Histomorphometry: the rate of bone formation and quantification of collagen types 1 and 3 

 Sections stained with Masson’s trichrome were analyzed under bright field microscopy, and sections stained with picrosirius red were analyzed under polarized light microscopy AxioImager^®^ Z2 (Zeiss, Germany). Both were quantified with Axiovision^®^ software (Zeiss, Germany). Images were captured using a digital camera (Zeiss^®^) with magnifications of 50× and 100×. The Masson’s trichrome staining was used to measure the rate of newly formed bone tissue, expressed as a percentage of the total callus area (B.Ar/T.Ar, %). The picrosirius red staining was used to calculate the area of collagen type 1 and 3 depositions, expressed by the total callus area (Col1.Ar/Tt.Ar, % and Col3.Ar/Tt.Ar, %).

###  RNA isolation and real-time PCR 

 Total RNA was extracted from the fracture callus (n=6 per group, 14 days post-fracture) using the SV Total RNA Isolation System (Promega, Madison, Wisconsin, USA) following the manufacturer’s instructions. Complementary DNA (cDNA) synthesis was performed with 1 μg RNA using the High Capacity cDNA Reverse Transcription Kit (Applied Biosystems, Foster City, CA, USA) following the manufacturer’s instructions. TaqMan^®^ gene expression assays (Applied Biosystems, USA) were used for quantifying *Collagen Type I Alpha 1 Chain* (*Col1a1)* (assay ID: Rn01463848_m1), *Runt Related Transcription factor 2* (*Runx2)* (Rn01512300_m1), *Osterix* (Rn02769744_s1), and *Sost* (Rn00577971_m1) expression by quantitative PCR on an StepOnePlus PCR machine (Applied Biosystems) and were normalized to the expression of the reference gene *Gapdh* (Rn01775763_g1). Samples were run in duplicate, and relative expression was calculated using 2^−ddCT^, the ddCt was calculated as dCt[goi_Res_ − ref_Res_] − dCt[goi_Con_ − ref_Con_] where *goi* is the gene of interest and *ref* is the reference gene. For descriptive and statistical analyses, ddCT was applied as a continuous variable. Minimum information for publication of quantitative real-time PCR experiments (MIQE) guidelines was followed for designing and interpreting the results of quantitative real-time PCR^21^.

###  Callus strength testing 

 After DXA and μCT assessments, both extremities of the fractured femurs were embedded in a cubic block of methyl methacrylate (Clássico^®^, Brazil). Care was taken to align the acrylic cement blocks and to avoid overheating during the cement setting (immersion in cold saline). Bones (n=7 animals per group) were hydrated and attached to a torsion machine (Instron 55MT^®^, USA) with a 2.0 Nm load cell and tested in torsion (anticlockwise rotation at 0.5°/sec) until failure. Femurs were kept wet with saline during the test. Using the specific software script, raw data were filtered, and torque at failure, energy, stiffness, and angular displacement at failure were obtained.

###  Statistical analysis 

 Continuous variables were expressed as means and standard deviations (SD). The results obtained in the groups were compared using t-Test where p-values less than 0.05 were considered statistically significant. All statistical analyses were performed with RStudio 1.0.153 (RStudio, Inc., USA).

## Results

###  Diet restriction on body mass 

**Figure 2:**
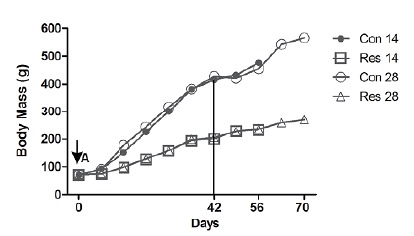
Comparison of body mass (g) variation among groups. **A**- At day 0 body mass was similar in both groups (p>0.05). Subsequently, the diet restricted rats showed a reduced gain of body mass. The vertical line indicates the establishment of the fracture (42 days). The 56th and 70th days represent the number of days of food restriction at euthanasia. There is a progressive difference between the normal diet and diet restricted groups. Con 14: control group followed by 14 days post-fracture; Res 14: dietary restriction group followed by 14 days post-fracture; Con 28: control group followed by 28 days post-fracture; Res 28: dietary restriction group followed by 28 days post-fracture (Data from the Laboratory of Bioengineering, School of Medicine of Ribeirao Preto, with permission).

 Upon study entry (day 0) there was no difference in body mass between groups (p>0.05, Fig. 2). As expected, dietary restriction rats gained much less weight than the controls on days 56 (231% versus 580%, p<0.05) and 70 (284% versus 707%, p<0.05).

###  Bone callus densitometry 

**Figure 3:**
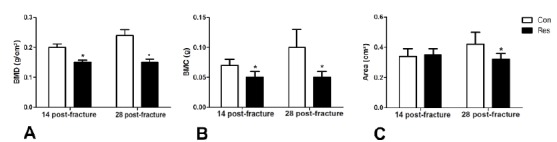
Dual-energy X-ray Absorptiometry (DXA) assessment. **A**- Comparison of bone mineral density (BMD) (g/cm^2^); **B**- Bone mineral content (BMC) (g) and **C**- The area (cm^2^) of the bone callus between groups at 14 and 28 days post-fracture. Asterisks indicate significant difference (p<0.05).

Figure 3 shows a comparison of BMD (3A), BMC (3B), and area (3C) of the bone callus for each group at both post-operative follow-ups. At 14 days post-fracture, dietary restricted rats had a significantly lower BMD and BMC than the controls (p<0.05). At 28 days post-fracture, all three parameters (BMD, BMC, and area) were lower in undernourished rats (p<0.05).

###  Bone callus microarchitecture 

**Figure 4:**
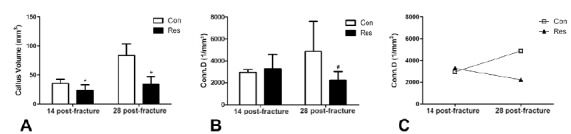
Graphic representation of the micro-computed tomography (μCT) parameters of the fracture callus. **A**- Callus volume: Dietary restriction resulted in decreased bone callus formation on both 14 and 28 days after fracture. **B**- Connectivity density of bone callus trabeculae. **C**- Comparison of the connectivity density of the callus at 14 and 28 days post-fracture. There is a clear divergence of the curves, demonstrating that connectivity increased (+66%) over time in control rats and decreased (by 31%) in the undernourished animals. Asterisks indicate significant difference (p<0.05) and hashtag indicates p=0.09.

 Dietary restriction reduced the callus volume by 34% on day 14 post-fracture and by 59% on day 28 (p<0.05, Fig. 4A). The trabecular connectivity density was reduced in the dietary restriction on day 28 after bone fracture (−54%, p=0.09). In addition, this parameter was increased by 66% in the control group on day 28 compared to day 14. Conversely, it decreased in the diet restrict group by 31% (Fig. 4 B,C) and may represent the main reason for the reduction in mechanical resistance against failure.

###  The rate of new bone callus formation and collagen quantity 

**Figure 5:**
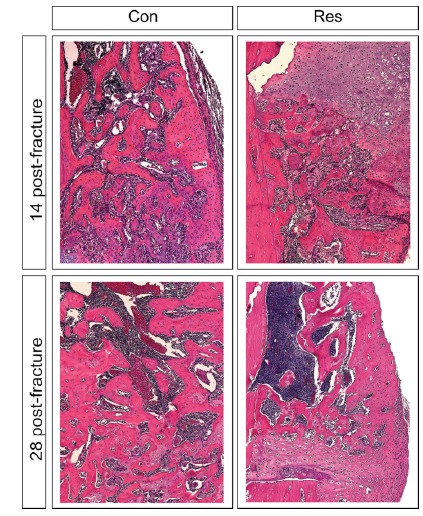
Histological aspects of the bone callus. Dietary restriction induced a delay in cell proliferation and differentiation during fracture healing. At 14 days after fracture, control rats exhibited a bone callus formed by trabeculae, while dietary restricted rats showed undifferentiated tissue and some cartilage tissue. The delay persisted and was incremented in the latest stage of healing, when control rats had very dense and thick trabeculae forming the callus and dietary restricted rats only showed thin and very spaced trabeculae (Hematoxylin and eosin, original magnification x100).

 At 14 days after bone fracture, control rats showed a bone callus formed by cartilaginous tissue and newly formed trabeculae (Fig. 5) with abundant collagen deposition. Conversely, dietary restricted rats exhibited callus mainly formed by undifferentiated tissue, cartilaginous tissue, and very few and spaced immature trabeculae (Fig. 5), associated with little collagen deposition. These qualitative findings were confirmed by histomorphometric assessment, given the rate of new bone formation (p=0.06) and the amount of both types of collagen were lower in the callus of dietary restricted rats (p<0.05). 

**Figure 6:**
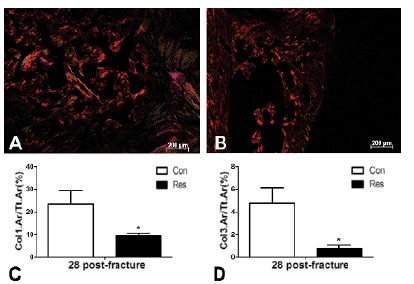
Polarized light image from the bone callus area. Type 1 collagen fibers are expressed in red-orange color and type 3 collagen fibers in yellowish-green. **A**- Control group, 28 days post-fracture. **B**- Dietary restriction group, 28 days post-fracture. Quantitative evaluation (%) of these images are reported in the graphs below, and are characterized by the decrease of the collagen type 1 (**C**) and collagen type 3 (**D**) fibers in the Res group. Asterisks indicate significant differences (p<0.05) (Picrosirus red, original magnification x100).

**Figure 7:**
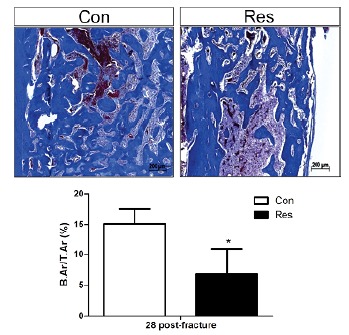
Bone callus 28 days post-fracture. The newly formed bone was significantly reduced in the dietary restricted rats and the degree of reduction is expressed quantitatively in the graph below (B.Ar/T.Ar, %). Asterisk indicates significant difference (p<0.05) (Masson’s trichrome, x100).

 This delay in the healing process was more marked on day 28 post-fracture. Control rats displayed a bone callus consisting of organized, thick, and dense trabeculae (Fig. 5), with abundant collagen deposition (Fig. 6). Dietary restricted rats only showed thin, unorganized, and disperse trabeculae associated with a large amount of cartilaginous tissue (Fig. 5) and limited collagen deposition (Fig. 6). The rate of bone callus formation remained lower in the group submitted to dietary restriction (Fig. 7) when compared to controls. Furthermore, undernutrition decreased the degree of types 1 and 3 collagen deposition (Fig. 6) in comparison to control rats (p<0.05).

###  Bone callus strength 

**Figure 8:**
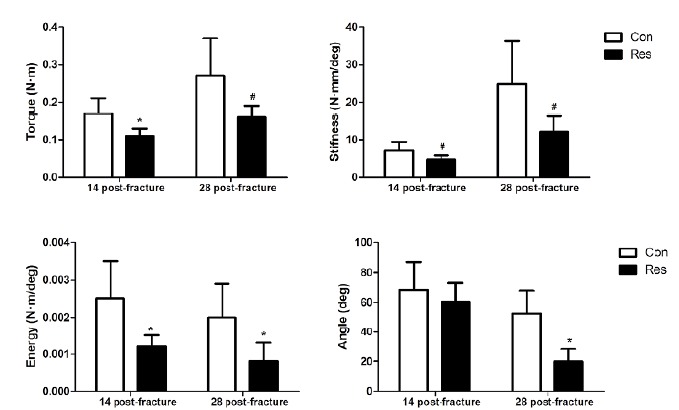
Parameters of the torsion test. The diet restricted animals showed smaller figures, which indicates a weakening of the bone (except for angle displacement at failure). Asterisks indicate significant differences (p<0.05) and hashtags indicate p=0.06.

Figure 8 shows the mechanical parameters of bone callus. On day 14 post-fracture, dietary restriction impaired the ability of bone callus to resist torque (p<0.05), stiffness (p=0.06), and energy (p<0.05). On day 28 post-fracture, dietary restriction not only impaired the ability of bone callus to resist torque (p=0.06), stiffness (p=0.06), energy (p<0.05), but it also reduced its capacity to tolerate angle displacement before failure (p<0.05), likely due to the lower collagen expression and deposition.

###  Gene expression 

**Figure 9:**
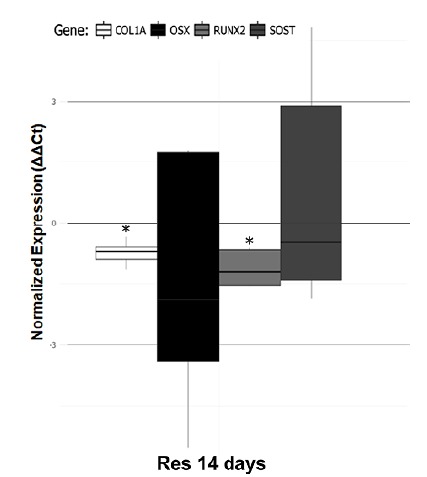
Gene expression in bone healing. Dietary restriction down-regulates the expression of osteoblast differentiation markers *Col1a1* and *Runx2* on day 14 after fracture. Asterisks indicate significant differences (p<0.05).

Figure 9 shows the relative gene expression via ddCT. The osteoblast differentiation markers *Col1a1* and *Runx2* were less expressed in dietary restricted rats when compared with control rats after 14 days of fracture (p<0.05). We did not find any difference regarding the osteocytes-related gene (*Sost*) and the gene *Osterix* also related to osteoblastic differentiation.

## Discussion 

 Considering that the peak bone mass achieved during skeletal growth is perhaps one of the most important risk factors for bone osteoporosis later in life^22^, any condition leading to a reduction in peak bone mass during the growth period should also be considered for its potential long-term effects. Malnutrition is one such condition that may occur at any stage of life and impairs bone quality^1^ ^,^ ^5^ ^,^ ^15^. 

 Devlin *et al*.^1^ studied the effects of caloric restriction on bone tissue and found an unbalanced bone turnover (decreased formation and increased resorption) and lower levels of insulin growth factor and leptin, leading to detrimental changes in both trabecular and cortical microstructure, thus decreasing mechanical strength. We hypothesize that these detrimental changes caused by undernutrition may also affect physiological fracture healing, as a coordinated activity by several cells and growth factors are needed to heal bone.

 After a bone is fractured, four overlapped phases occur: inflammation, soft callus formation, hard callus formation, and remodeling^17^ ^,^ ^23^. During the inflammatory stage, a hematoma forms between bone fragments creating a microenvironment that releases multiple growth factors and cytokines^23^ ^,^ ^24^. Subsequently, immature mesenchymal cells are differentiated into chondroblasts and osteoblasts. The differentiation, proliferation and maturation of chondroblasts lead to the formation of cartilage (soft callus)^25^Once the cartilage matrix is formed, its mineralization occurs causing apoptosis and triggering osteogenesis, thus constituting the primary osseous structure (hard callus). The remodeling phase is a long-term event responsible for reshaping the initial bone callus (woven bone) to recover bone function to the pre-fracture level before the fracture occurred^26^. This is a long-term process in which the mechanical forces are essential for reabsorbing and redirecting the callus trabeculae.

 Although it has been previously documented that the body maintains priority for growth even under extreme conditions of cessation of body weight gain^27^, we still believe that bone healing is highly impaired by dietary restriction. Some authors have studied the effects of depletion of specific nutrients on bone fracture healing, but none have examined the restriction of intake of the whole diet. Thus, herein, we investigated the effects of dietary restriction on bone fracture healing simulating conditions that commonly occur in many areas across the world.

 Our results indicated that dietary restriction during skeletal growth significantly impaired bone fracture healing. The detrimental phenotype changes observed in the bone callus of food-restricted rats may be partially explained by the lower gene expression of Col1a1 and Runx2. The essential role of type 1 collagen on endochondral bone formation is well documented in the literature^28^. Our histomorphometric findings corroborated our gene expression analysis by evidencing the lower deposition of collagen at the bone callus in rats under dietary restriction. In addition, these animals also showed a decrease in collagen type 3 depositions at the bone callus. In a previous study, it has been shown that the presence of collagen type 3 in the mesenchymal condensations precedes chondrogenesis and osteogenesis^29^. In our model, food restriction impaired both types of collagen, those found in mature bone and those in newly formed bone. The expression of Runx2 is essential for bone callus formation since it has a crucial role in controlling osteoblast differentiation and skeletal development^30^ ^,^ ^31^. Taken together, these changes in gene expression due to dietary restriction resulted in fracture healing delay mostly caused by an impairment in bone formation (chondroblastogenesis and osteoblastogenesis). On day 14 after the fracture, the bone callus of dietary restricted rats was mainly formed by cartilaginous and undifferentiated tissue, with very few and very thin trabeculae. The delay persisted and was more intense on day 28 with the callus still formed by thin and disperse trabeculae. At this stage, the bone callus was expected to be formed by thick and dense trabeculae. Consequently, the bone callus of dietary restricted rats was less dense and weaker. 

 To our knowledge, this study is the first to investigate the effects of global dietary restriction on bone healing. In conclusion, a significant delay in osseous healing was evidenced due to a severe reduction in bone formation caused by dietary restriction. Our results highlighted several molecular disruptions, which affected the well-orchestrated cascade of biological events that occur during fracture healing and resulted in severe phenotype changes. Considering that clinical undernutrition may occur at any stage of life, special care should be provided for those sustaining a concomitant bone fracture due to the high probability of delayed and disrupted osseous union.
